# Supplementary material for: Differential antitumor activity of compounds targeting the ubiquitin-proteasome machinery in gastrointestinal stromal tumor (GIST) cells
Source: Sci Rep. 2020 Mar 20;10:5178. doi: 10.1038/s41598-020-62088-7 (PMC7083865; doi:10.1038/s41598-020-62088-7)
Supplement: Supplementary file 1 — Supplementary Methods and Figures. [file 41598_2020_62088_MOESM1_ESM.pdf]

SUPPLEMENTARY INFORMATION

Differential antitumor activity of compounds targeting the ubiquitin-proteasome machinery in gastrointestinal stromal tumor (GIST) cells

Jessica L. Rausch, Areej A. Ali, Donna M. Lee, Yemarshet K. Gebreyohannes, Keith R. Mehalek, Aya Agha, Sneha S. Patil, Yanis Tolstov, Jasmien Wellens, Harbir S. Dhillon, Kathleen R. Makielski, Maria Debiec-Rychter, Patrick Schöffski, Agnieszka Wozniak, Anette Duensing

SUPPLEMENTARY METHODS

Immunological and cell staining methods

Protein lysates of cells growing as monolayer were prepared by scraping cells into lysis buffer (1% NP-40, 50 mM Tris-HCl pH 8.0, 100 mM sodium fluoride, 30 mM sodium pyrophosphate, 2 mM sodium molybdate, 5 mM EDTA, 2 mM sodium orthovanadate) containing protease inhibitors (10 µg/ml aprotinin, 10 µg/ml leupeptin, 1 µM phenylmethylsulfonyl fluoride). Lysates were incubated for 1 h with shaking at 4°C and then cleared by centrifugation for 30 min at 14,000 rpm (4°C). Protein concentrations were determined using the Bradford assay (Biorad). Thirty µg of protein were loaded on a 4-12% Bis-Tris gel (Invitrogen) and blotted onto a nitrocellulose membrane.

For immunofluorescence analysis, cells grown in chamber slides (BD Biosciences) were briefly washed in PBS and fixed in 4% paraformaldehyde in PBS for 15 min at room temperature (RT). Cells were then washed in PBS and permeabilized with 1% Triton-X 100 in PBS for 15 min (RT) followed by washing in PBS and blocking with 10% normal donkey serum (Jackson Immunoresearch) for 15 min. Primary antibody incubation was done overnight at 4°C in a humidified chamber and an additional hour at 37°C the next morning. After a brief wash in PBS, cells were incubated with Alexa Fluor 488-conjugated goat anti-rabbit secondary antibodies (Invitrogen; 30 min, RT), washed (PBS) and counterstained with 4',6-diamidino-2-phenylindole (DAPI; Vector Laboratories). Cells were analyzed using an Olympus AX70 epifluorescence microscope equipped with a SPOT RT digital camera and SPOT imaging software.

Immunohistochemistry was performed on sections (4µm) of formalin-fixed, paraffin-embedded mouse tumors from *in vivo* experiments. Slides were deparaffinized, underwent antigen retrieval and were incubated with rabbit monoclonal primary antibodies. The signal was detected using anti-rabbit EnVision+ System-HRP secondary

antibodies and 3’diaminobenzidine-tetrahydrochloride (DAB; both from Dako/Agilent). Apoptotic and mitotic cells were visualized by immunohistochemical staining for cleaved PARP and phosphorylated histone H3 S10, respectively, and Ki-67 was used to evaluate cellular proliferation[35]. The number of positive cells was counted per 10 high power fields (HFP) at 400-fold magnification.

Detection of apoptotic cells *in vitro* was done using the In situ Cell Death Detection Kit (Roche Applied Sciences) according to manufacturer’s recommendations.

Luminescence-based proliferation and apoptosis assays

Cell viability and apoptosis studies were performed using the CellTiter-Glo (readout: ATP) and Caspase-Glo (readout: caspase 3/7 activity) luminescence-based assays (Promega). Cells were plated in 96-well flat-bottomed plates (Perkin Elmer), cultured for 24 h and then incubated for 48 h (Caspase-Glo) or 72 h (CellTiter-Glo) with the respective compounds at indicated concentrations or DMSO-only solvent control. Luminescence was measured with a BioTek Synergy 2 Luminometer (BioTek). Data were normalized to the DMSO-only control group.

Reverse transcriptase (RT)-PCR and quantitative real time RT-PCR

RT-PCR and quantitative real time RT-PCR (qRT-PCR) were performed as described previously[5]. Exon-overlapping, mRNA/cDNA-specific primers were used to amplify *KIT* (forward: 5’-TCATGGTCGGATCACAAAGA-3’, reverse: 5’-AGGGGCTGCTTCCTAAAGAG-3’; Operon) and *β-actin* (forward: 5’-CCAAGGCCAACCGCGAGAAGATGAC-3’, reverse: 5’-AGGGTACATGGTGGTGCCGCCAGAC-3’). *β-actin* served as reference gene for relative quantification in quantitative real time RT-PCR (qRT-PCR) experiments.

SUPPLEMENTARY FIGURE LEGENDS

Supplementary Figure S1. Delanzomib is highly effective in IM-sensitive and IM-resistant GIST cells and rapidly leads to a dose- and time-dependent accumulation of ubiquitinated proteins.

(A) Immunoblot analysis for markers of cell cycle regulation and apoptosis after treatment with increasing concentrations (0.0001 μM – 10 μM) of delanzomib (DLZ). Grouped immunoblot images are either cropped from different parts of the same gel or from a separate gel run with another aliquot of the same protein lysate.

(B) Dose-dependent effect of delanzomib on induction of apoptosis in IM-sensitive GIST-T1 and IM-resistant GIST430 cells as measured by TUNEL assay. Graphs represent mean and standard error of at least three experiments with at least 100 cells counted each. **, $p \leq 0.01$ in comparison to control; ***, $p \leq 0.001$ in comparison to control (Student's t-test, 2-tailed).

(C) Immunoblot analysis for markers of cell cycle regulation and apoptosis after treatment of IM-sensitive (GIST882) and IM-resistant (GIST430) cells with DMSO or delanzomib (0.1 μM) for the indicated times.

(D, E) Dose- **(D)** and time-dependent **(E)** accumulation of mono-ubiquitinated proteins in IM-sensitive (GIST-T1) and IM-resistant (GIST48) cells after delanzomib treatment as determined by immunoblotting. As expected, kinase inhibitor treatment with imatinib (IM) or sunitinib (SU; both 1.0 μM) did not lead to increased ubiquitination when compared to DMSO-treated controls. IM and SU serve as standard treatment controls for IM-naïve and IM-resistant cell lines, respectively. Bortezomib (BO) serves as control for proteasome inhibition.

Supplementary Figure S2. Carfilzomib (CFZ) and ixazomib (IXA) lead to cell cycle exit and apoptosis in IM-sensitive and IM-resistant GIST cells.

(A–C) Dose-dependent effect of carfilzomib and ixazomib on cell cycle exit and induction of apoptosis as measured by immunoblotting **(A)** and TUNEL assay **(B, C)**. Graphs represent mean and standard error of at least three experiments with at least 100 cells counted each. *, $p \leq 0.05$ in comparison to control; **, $p \leq 0.01$ in comparison to control; ***, $p \leq 0.001$ in comparison to control (Student's t-test, 2-tailed).

(D, E) Time-dependent effect of carfilzomib **(D)** and ixazomib **(E)** on cell cycle exit and induction of apoptosis of GIST cells as determined by immunoblotting. Treatment with carfilzomib (1.0 µM) **(D)** or ixazomib (0.1 µM) **(E)**, respectively, for the indicated times.

(A, D, E) Grouped immunoblot images are either cropped from different parts of the same gel or from a separate gel run with another aliquot of the same protein lysate.

Supplementary Figure S3. Carfilzomib (CFZ) and ixazomib (IXA) lead to a dose- and time-dependent accumulation of ubiquitinated proteins, but to a lesser extent than delanzomib.

(A, B) Dose- **(A)** and time-dependent **(B)** accumulation of mono-ubiquitinated proteins in GIST cells after carfilzomib treatment (0.0001 µM – 10 µM, **(A)**; 1.0 µM **(B)**) as determined by immunoblotting. As expected, kinase inhibitor treatment with IM or SU (both 1.0 µM) did not lead to increased ubiquitination when compared to DMSO-treated controls. Note that the effect seems to be transient and early effects may have been missed in the 72 h-treated dose-response shown in **(A)**. IM and SU serve as standard treatment controls for IM-naïve and IM-resistant cell lines, respectively. Bortezomib (BO) serves as control for proteasome inhibition.

(C, D) Dose- **(C)** and time-dependent **(D)** accumulation of mono-ubiquitinated proteins in GIST cells after ixazomib treatment (0.0001 µM – 10 µM, **(C)**; 0.1 µM **(D)**) as determined by immunoblotting. As expected, kinase inhibitor treatment with IM or SU (both 1.0 µM; 72 h) did not lead to increased ubiquitination when compared to DMSO-treated controls.

Supplementary Figure S4. The DUB inhibitor b-AP15 and the NAE inhibitor MLN4924 are not as effective as 26S proteasome inhibitors.

(A, B) Immunoblot analysis for markers of cell cycle regulation and apoptosis in GIST cells after treatment with increasing concentrations (0.0001 µM – 10 µM; 72 h) of b-AP15 **(A)** or MLN4924 **(B)**. Treatment with IM or SU (both 1.0 µM) serve as standard treatment controls for IM-naïve and IM-resistant cell lines, respectively. Grouped immunoblot images are either cropped from different parts of the same gel or from a separate gel run with another aliquot of the same protein lysate.

Supplementary Figure S5. Delanzomib (DLZ) treatment leads to upregulation of soluble histone H2AX and transcriptional downregulation of KIT expression.

(A, B) Immunoblot analysis of GIST-T1 and GIST430 cells treated with delanzomib at the indicated concentrations for 72 h **(A)** or with 0.1 µM delanzomib for the indicated times **(B)** and probed for phospho-H2AX (S139) and total H2AX as well as phospho-KIT (Y719) and total KIT. Grouped immunoblot images are either cropped from different parts of the same gel or from a separate gel run with another aliquot of the same protein lysate.

(C) Immunofluorescence microscopic analysis of GIST-T1 and GIST430 cells treated with DMSO or 0.1 µM delanzomib for 72 h and stained for the transcriptional co-activator CREB-binding protein (CBP; green). Nuclei were stained with DAPI. Scale bar, 20 µm.

Supplementary Figure S6. Carfilzomib and ixazomib lead to upregulation of soluble histone H2AX and transcriptional downregulation of KIT expression, similar to delanzomib.

(A – D) Immunoblot analysis of GIST cells treated with carfilzomib **(A, B)** or ixazomib **(C, D)** at the indicated concentrations for 72 h **(A, C)** or with 1.0 μM carfilzomib **(B)** or with 0.1 μM ixazomib **(D)** for the indicated times and probed for phospho-H2AX (S139) and total H2AX as well as phospho-KIT (Y719) and total KIT.

(E, F) Immunofluorescence microscopic analysis of GIST cells treated with DMSO or 1.0 μM carfilzomib **(E)** or 0.1 μM ixazomib **(F)** for 48 h and stained for the transcriptional co-activator CREB-binding protein (CBP; green). Nuclei were stained with DAPI. Scale bar, 20 μm.

(A – D) Grouped immunoblot images are either cropped from different parts of the same gel or from a separate gel run with another aliquot of the same protein lysate.

Supplementary Figure S7. Re-distribution of transcriptional co-factor CREB binding protein (CBP) staining after delanzomib treatment over time.

Immunofluorescence microscopic analysis of GIST882 cells treated with DMSO or 0.1 μM delanzomib for up to 72 h and stained for CBP (green). Nuclei were stained with DAPI. Note CBP redistribution into large nuclear displacement foci starting at 24 h and complete loss at later time points. Scale bar, 20 μm.

Supplementary Figure S8. Delanzomib has *in vivo* antitumor activity in GIST xenografts.

(A) Histopathologic and immunohistochemical response of imatinib-resistant UZLX-GIST9 xenografts after 24 h to a one-time bolus treatment with delanzomib in comparison with placebo. Hematoxylin and eosin (H&E) as well as immunohistochemical staining for Ki-67 or cleaved PARP (10X magnification). Histopathologic response grade was determined according to Agaram et al.[56]. Data shown in graph represent at least ten tumors per group. Quantification of proliferation index (% Ki-67 positive cells) and percentage of cleaved PARP-positive cells (brown staining, respectively) represents the average of at least nine tumors per group. Columns, mean + SE; *, p≤0.05, **, p≤0.01 in comparison to control (Student's t-test, 2-tailed).

(B) Waterfall plot of best response of percent change of UZLX-GIST1 xenografts during a 21-day treatment with vehicle (black bars), delanzomib (white bars) or imatinib (grey bars).

(C) Spider plot of relative tumor volume of GIST430 xenografts treatment with vehicle or delanzomib over the course of a 21-day. *Placebo*, black lines; *delanzomib*, grey dotted lines.

C) Histopathologic response of imatinib-resistant GIST430 xenografts to treatment with delanzomib in comparison with placebo. Histopathologic response grade was determined according to Agaram et al.[56]. Data shown in graph represent at least eight tumors per group. Columns, mean + SE; *, p≤0.05 in comparison to control (Student's t-test, 1-tailed).

(D) Reduced mitotic activity of GIST430 xenografts after treatment with delanzomib in comparison with placebo. Quantification of mitotic cells (pH3-positive cells per 10 HPF; brown staining) represents the average of at least eight tumors per group. Columns, mean + SE; ***, p≤0.001 in comparison to control (Student's t-test, 2-tailed).

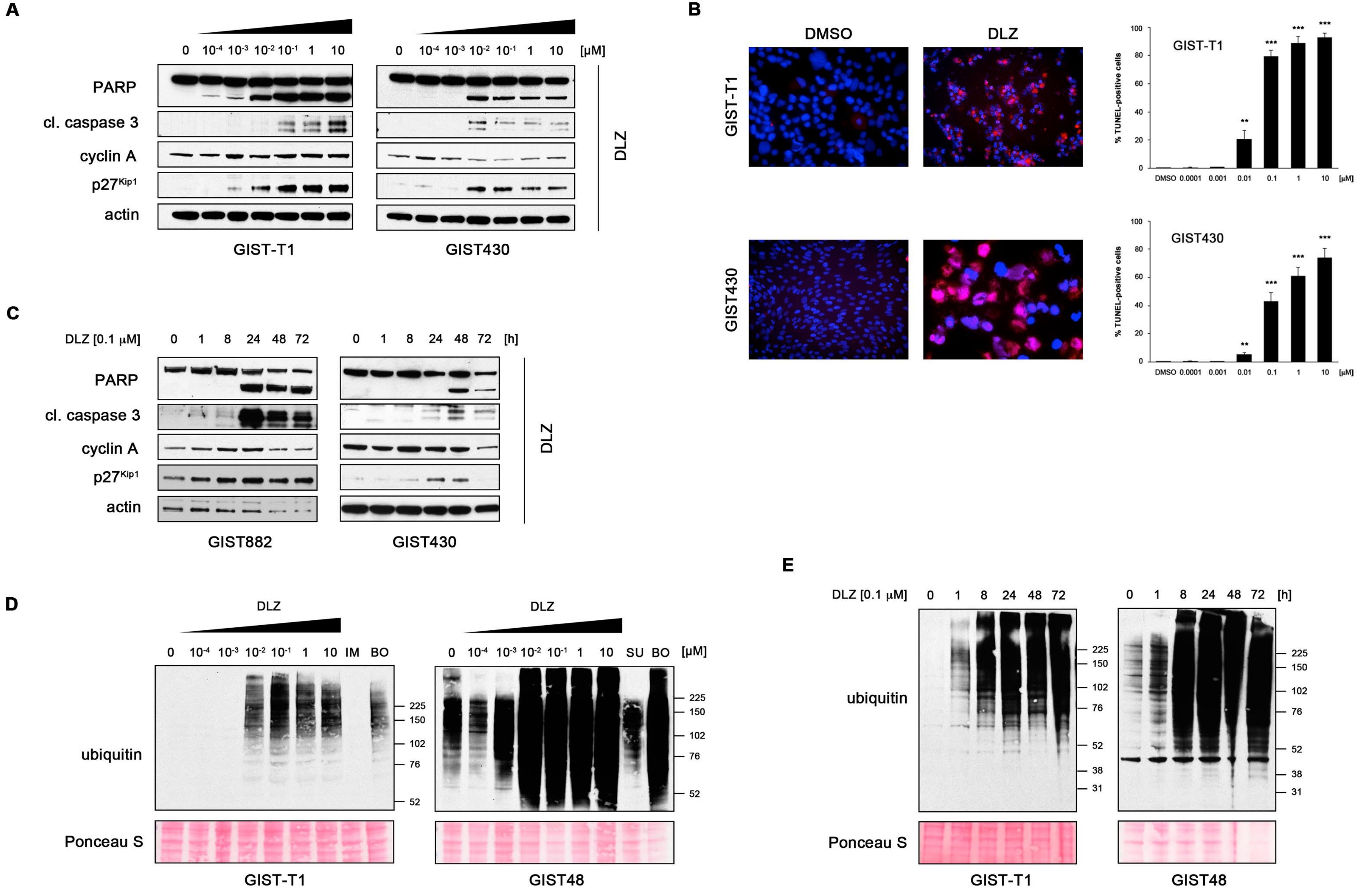

Rausch *et al.*, Supplemental Figure 1

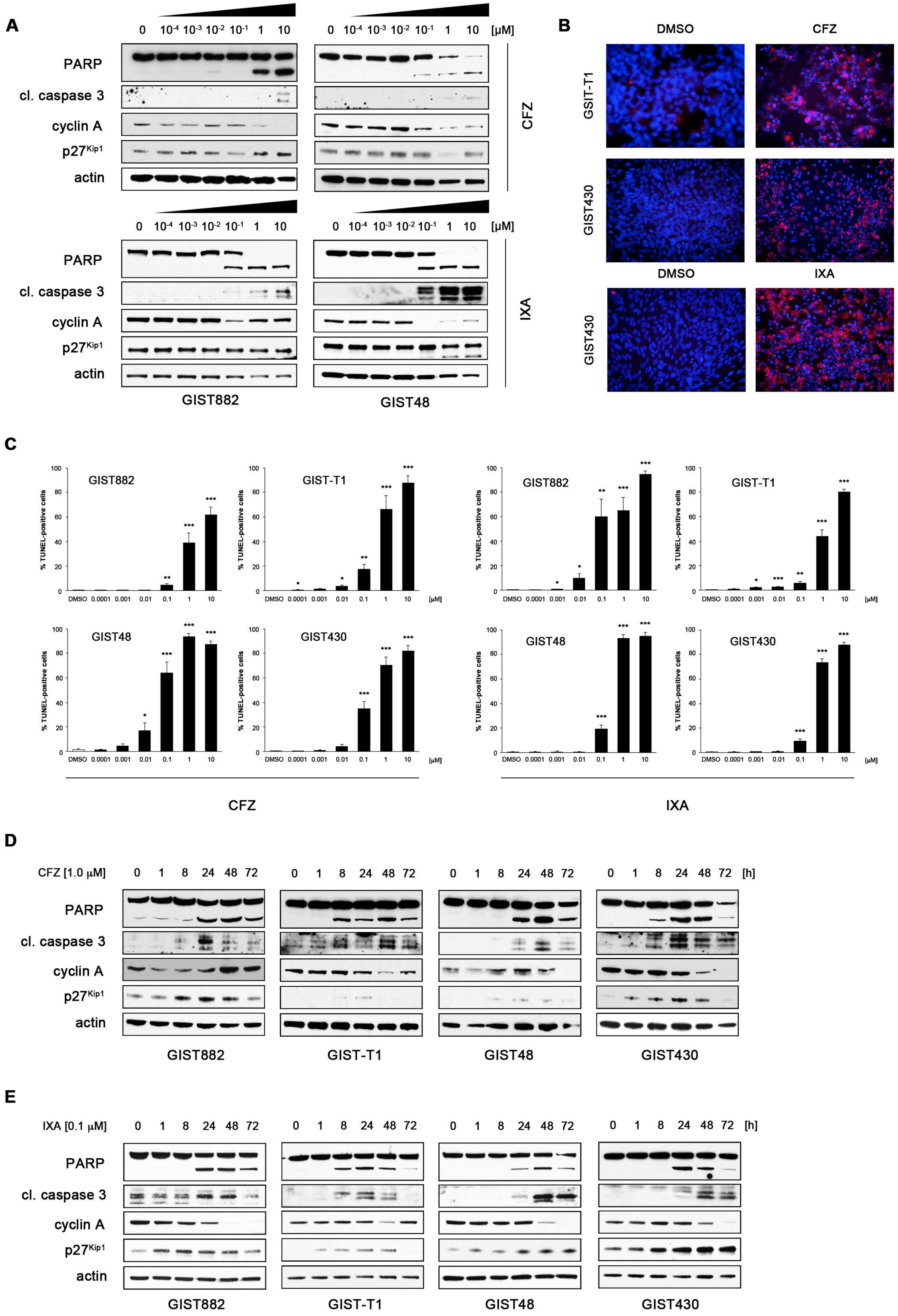

Rausch *et al.*, Supplemental Figure 2

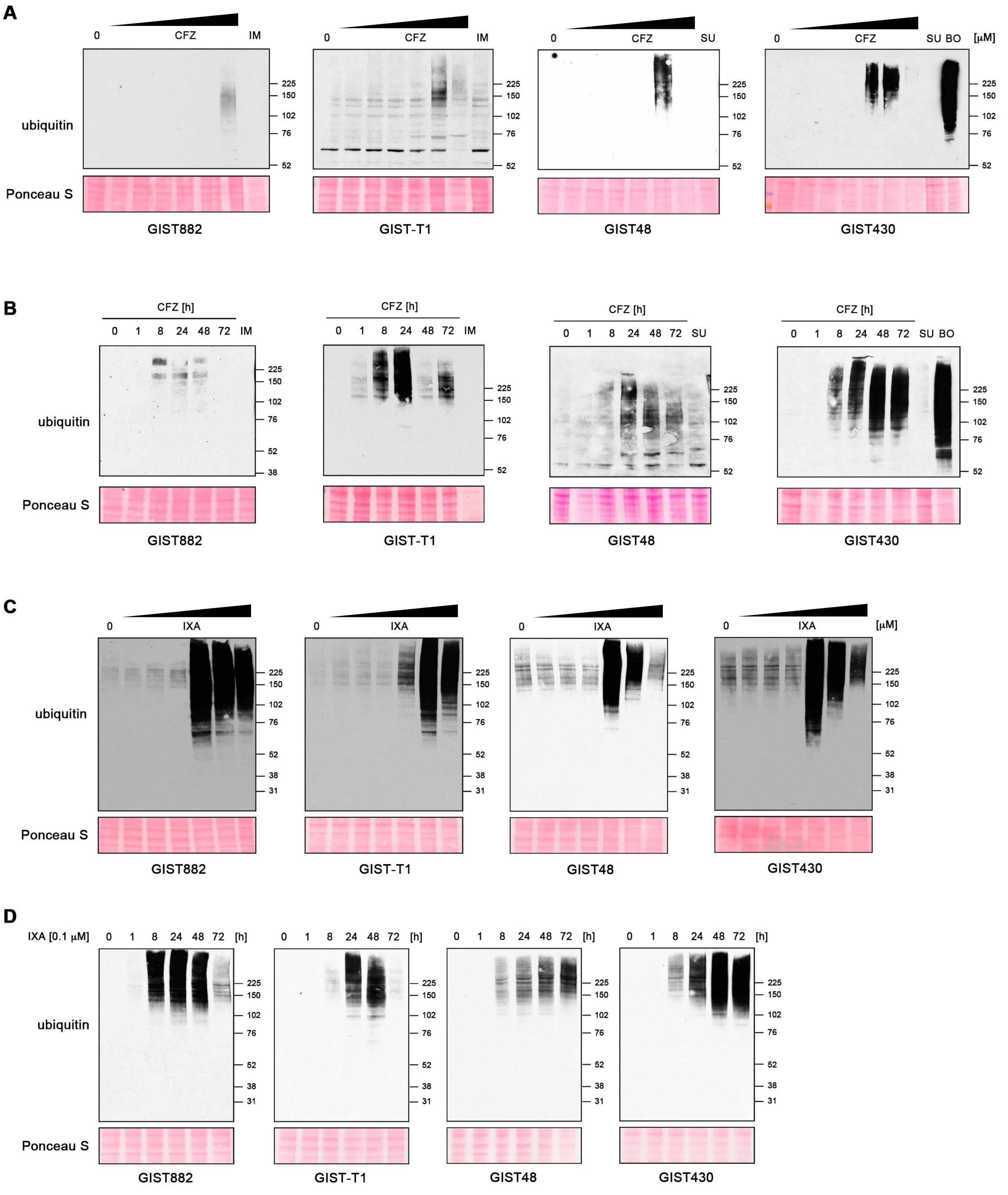

Rausch *et al.*, Supplemental Figure 3

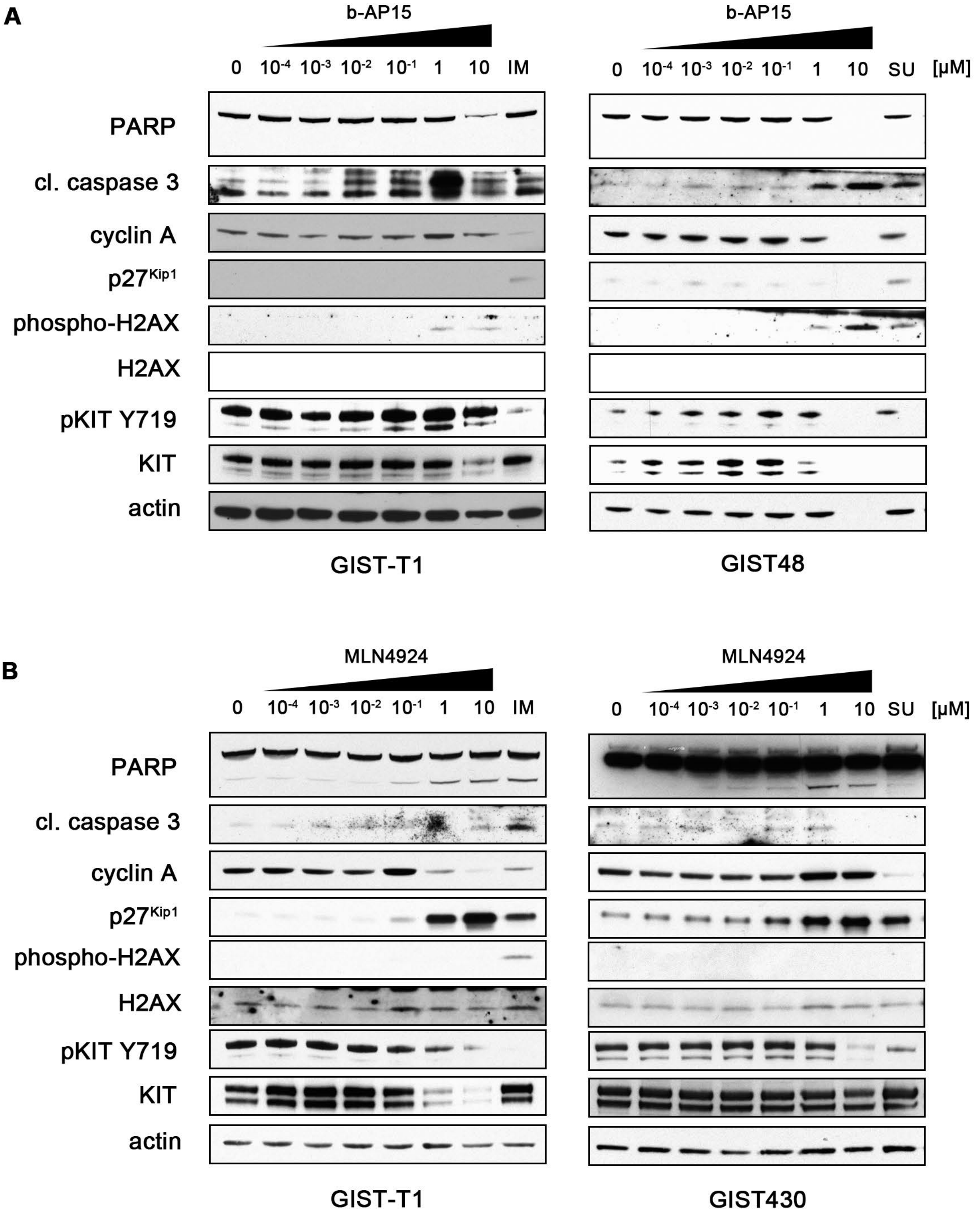

Rausch *et al*., Supplemental Figure 4

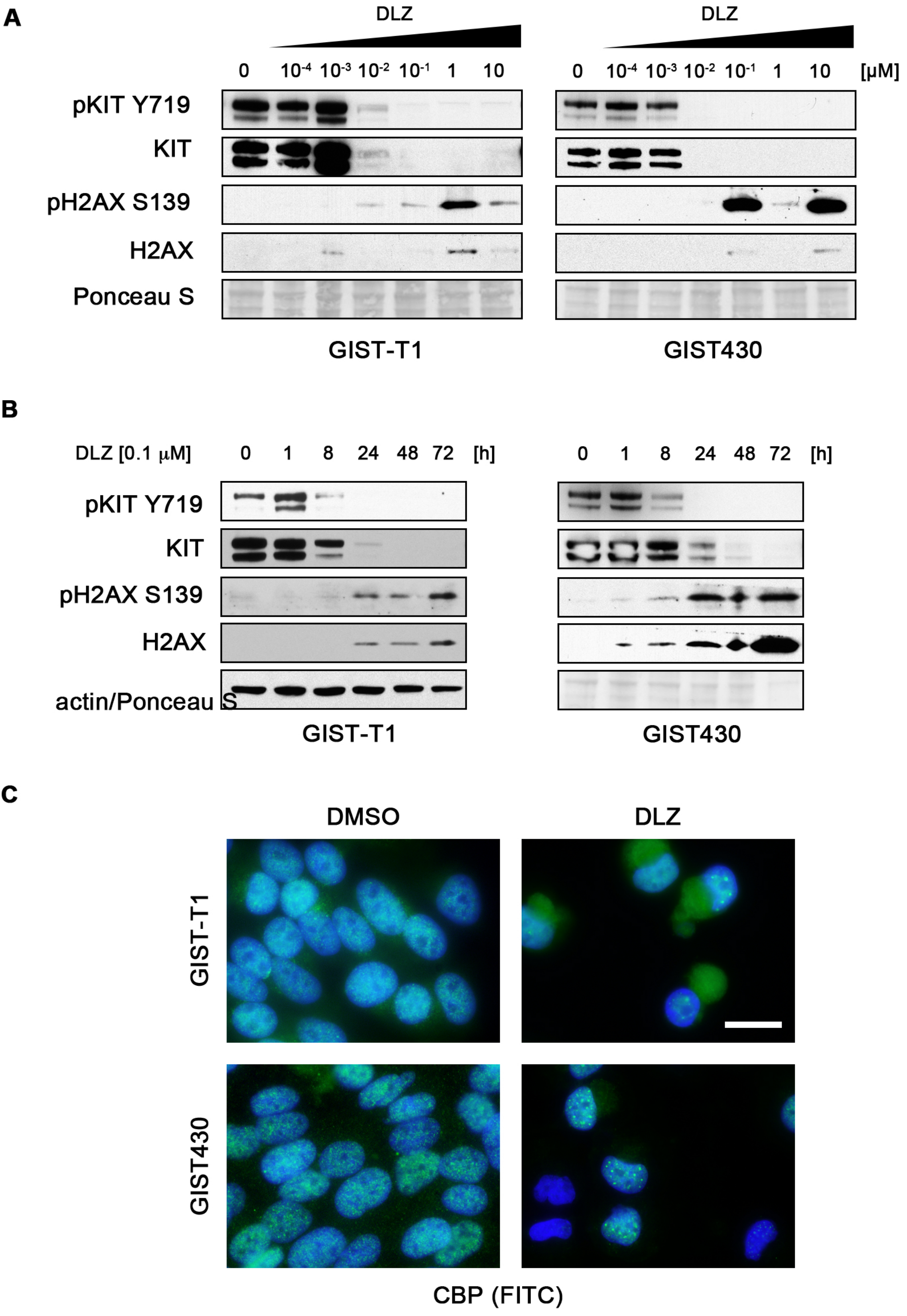

Rausch *et al.*, Supplemental Figure 5

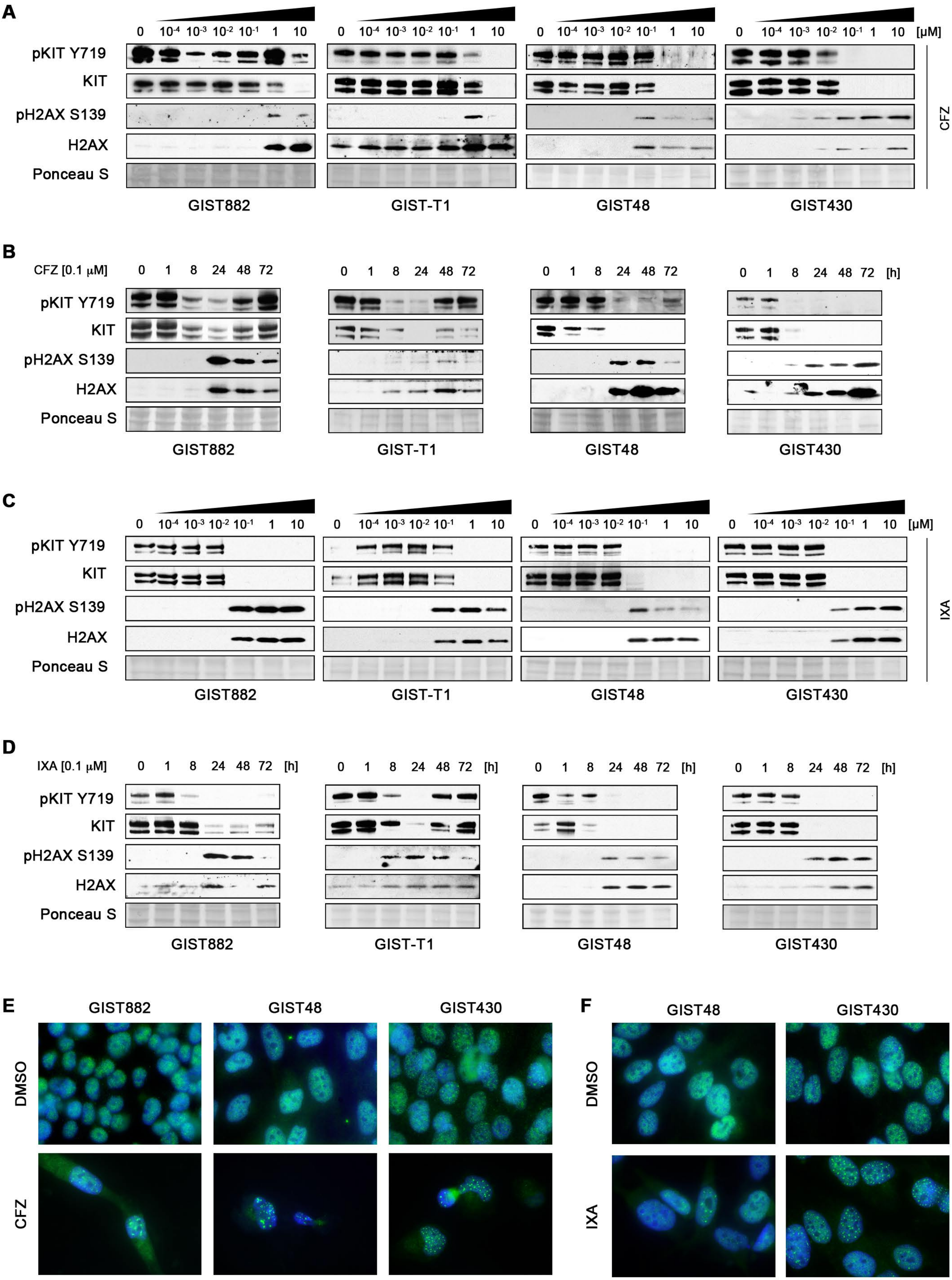

Rausch *et al.*, Supplemental Figure 6

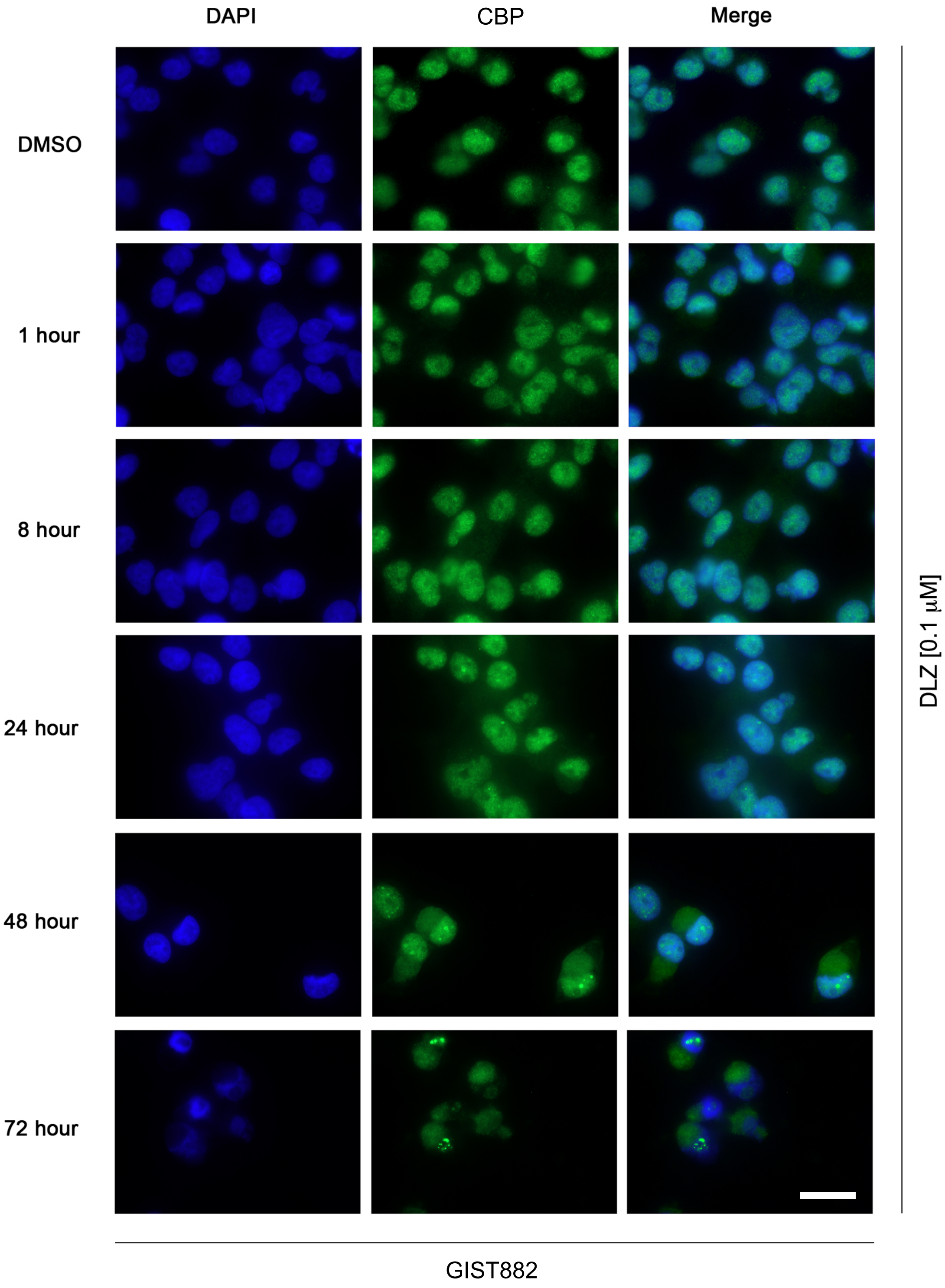

Rausch *et al.*, Supplemental Figure 7

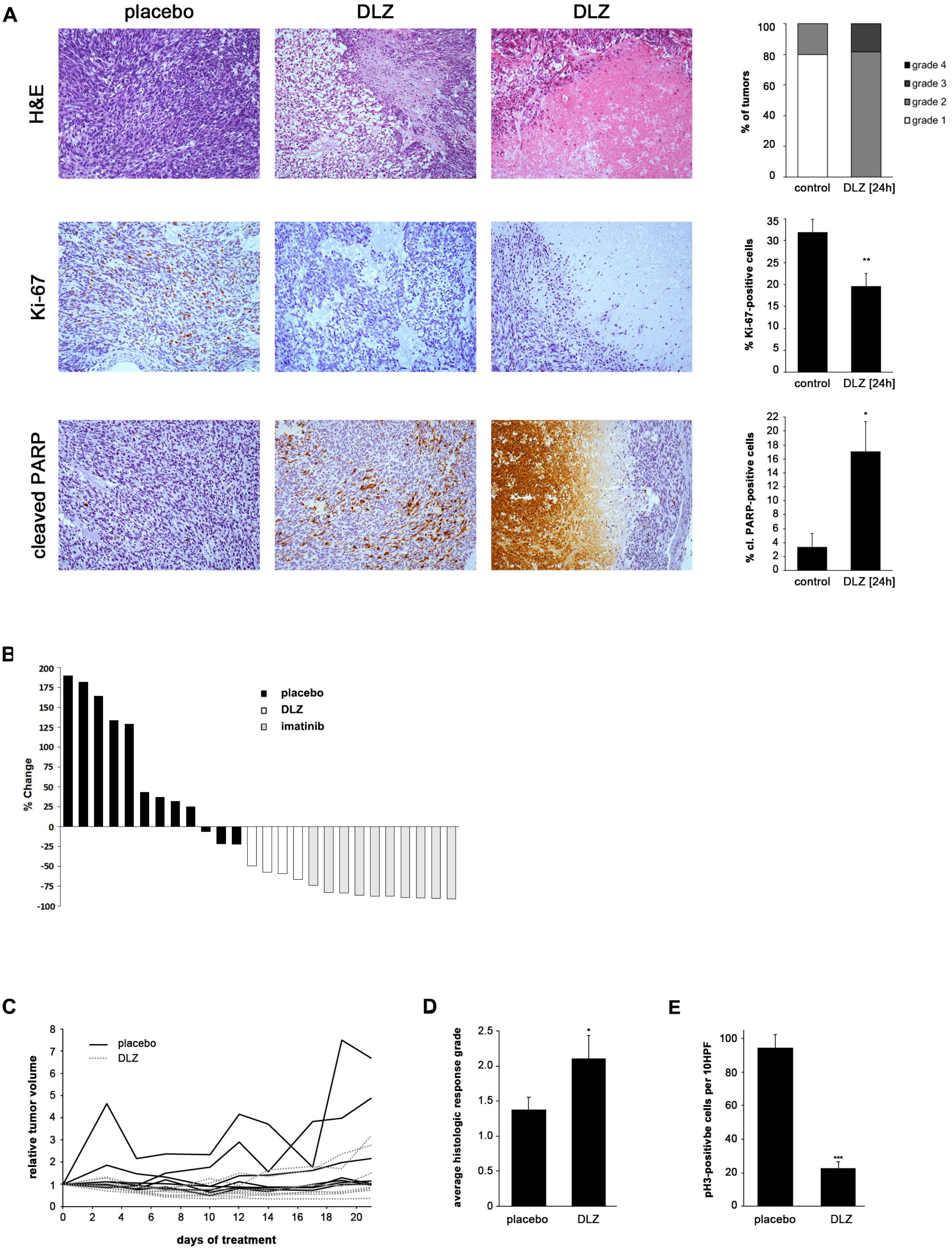

Rausch *et al.*, Supplemental Figure 8
